# Blood-Based β-Amyloid and Phosphorylated Tau (p-Tau) Biomarkers in Alzheimer’s Disease: A Systematic Review of Their Diagnostic Potential

**DOI:** 10.7759/cureus.79881

**Published:** 2025-03-01

**Authors:** Meghana Dasari, Joel Abraham Kurian, Sumanth Gundraju, Aishwarya Raparthi, Rooth V Medapati

**Affiliations:** 1 Department of General Medicine, Rangaraya Medical College, Dr. Nandamuri Taraka Rama Rao (NTR) University of Health Sciences, Kakinada, IND; 2 Department of General Medicine, Andhra Medical College, Dr. Nandamuri Taraka Rama Rao (NTR) University of Health Sciences, Kakinada, IND; 3 Department of Human Genetics, Andhra University, Visakhapatnam, IND

**Keywords:** alzheimer’s dementia, blood biomarkers, dementia, diagnosis, phosphorylated tau, prisma, systematic review, β-amyloid

## Abstract

Alzheimer’s disease (AD) is a progressive neurodegenerative disorder characterized by cognitive decline and neuropathological features such as amyloid-β (Aβ) plaques and phosphorylated tau (p-Tau) tangles. Blood-based biomarkers of Aβ and p-Tau have emerged as promising tools for early diagnosis, monitoring, and risk stratification of AD. This systematic review evaluates current evidence on the diagnostic utility of Aβ and p-Tau blood biomarkers in AD. This systematic review followed the Preferred Reporting Items for Systematic Reviews and Meta-Analyses (PRISMA) guidelines. A comprehensive literature search was conducted across PubMed, Scopus, and Web of Science for studies published between 2011 and 2024. This review synthesizes findings from 33 peer-reviewed studies to evaluate the diagnostic and prognostic utility of these biomarkers. Results demonstrate that blood Aβ and p-Tau levels strongly correlate with cerebrospinal fluid (CSF) biomarkers and neuroimaging measures of AD pathology. Among the biomarkers analyzed, p-Tau (including p-Tau181 and p-Tau217) consistently exhibited superior diagnostic accuracy, particularly in distinguishing AD from mild cognitive impairment (MCI) and cognitively normal individuals. The combination of Aβ and p-Tau biomarkers further improved diagnostic precision, supporting their complementary roles in AD pathology detection. Despite promising findings, significant heterogeneity among studies underscores the need for assay standardization, validation in diverse populations, and longitudinal research to establish clinical utility. This study concludes that blood-based Aβ and p-Tau biomarkers represent a significant advance in AD diagnostics, offering non-invasive, cost-effective, and scalable solutions for early detection and therapeutic monitoring.

## Introduction and background

Alzheimer’s disease (AD) is the most common form of dementia, affecting more than 55 million people globally, a number projected to triple by 2050 due to aging populations. This neurodegenerative disorder is characterized by cognitive decline, memory impairment, and neuropathological hallmarks, including extracellular amyloid-β (Aβ) plaques and intracellular neurofibrillary tangles of phosphorylated tau (p-Tau). Early and accurate diagnosis of AD is crucial for implementing therapeutic interventions and improving patient outcomes, yet current diagnostic methods, such as cerebrospinal fluid (CSF) biomarkers, positron emission tomography (PET) imaging, and neuropsychological assessments, are often invasive, expensive, and inaccessible to many patients.

In recent years, blood-based biomarkers have emerged as a promising alternative to traditional diagnostic methods. These biomarkers offer a minimally invasive, cost-effective, and scalable approach for identifying and monitoring AD pathology. Among the most studied blood biomarkers are Aβ, a key component of amyloid plaques, and p-Tau, a major contributor to neurofibrillary tangles. Both are integral to the amyloid and tau hypotheses of AD pathogenesis, which postulate that Aβ accumulation initiates the disease process and p-Tau aggregation drives neurodegeneration.

This review evaluates the diagnostic and prognostic utility of blood-based Aβ and p-Tau biomarkers in AD by synthesizing data from 33 peer-reviewed studies [1-38] published between 2011 and 2024. The primary objectives are to determine the accuracy of these biomarkers in differentiating AD from other cognitive conditions, examine their correlation with established CSF and imaging biomarkers, and explore their potential for integration into clinical workflows. By addressing these objectives, this study aims to contribute to the development of accessible diagnostic tools that facilitate early detection and improve patient outcomes in AD.

## Review

Methodology

Study Design

This systematic review was conducted following the Preferred Reporting Items for Systematic Reviews and Meta-Analyses (PRISMA) guidelines to ensure a transparent and comprehensive synthesis of the literature on blood biomarkers β-amyloid (Aβ) and phosphorylated tau (p-Tau) in the early diagnosis of AD.

**Figure 1:**
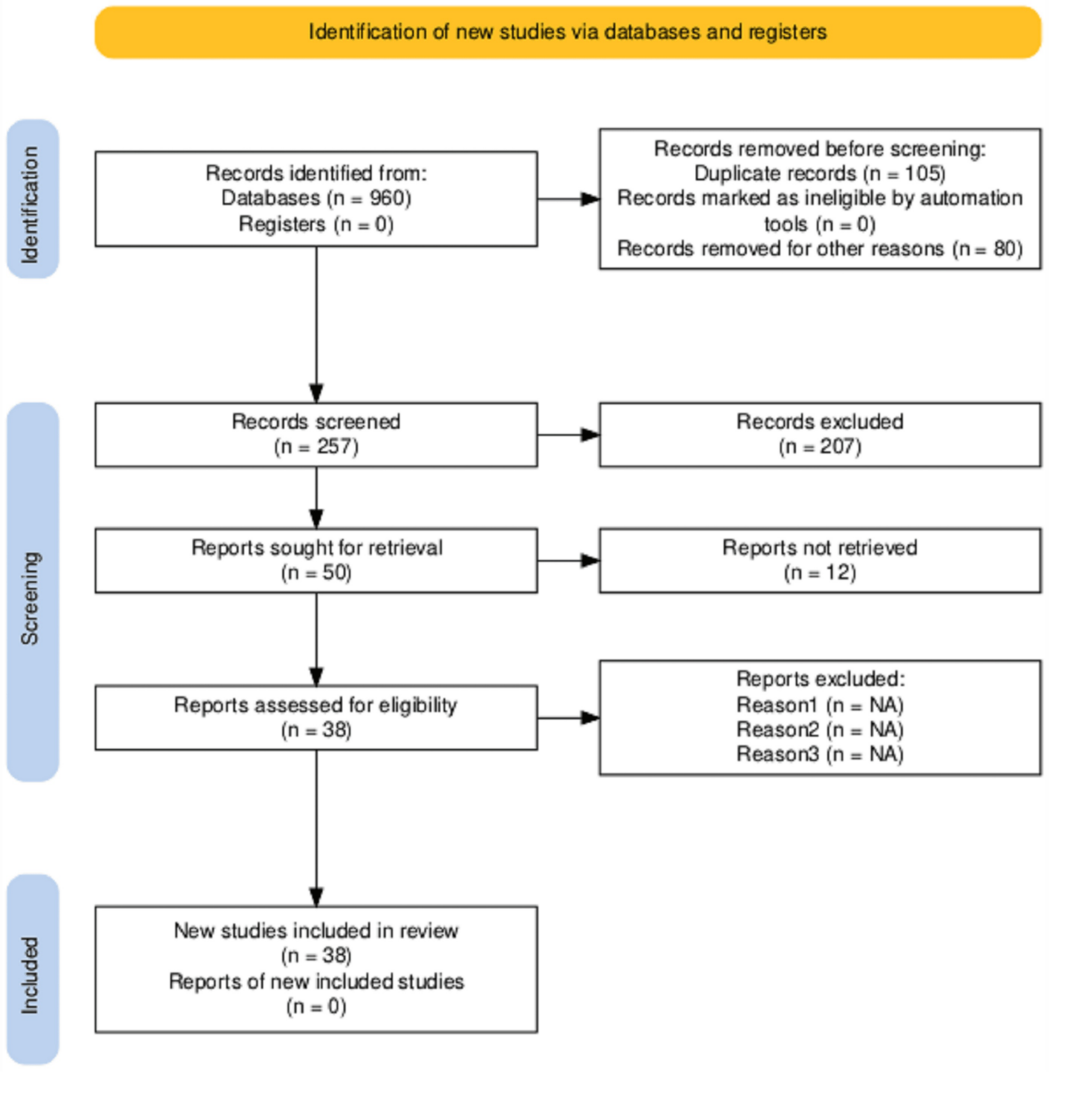
Preferred Reporting Items for Systematic Reviews and Meta-Analyses (PRISMA) flow diagram for study selection

Search strategy: A comprehensive search was performed in the following electronic databases: PubMed, Scopus, Web of Science, and Embase. The search covered studies published between 2011 and 2024 using predefined keywords and Medical Subject Headings (MeSH) terms, including "Alzheimer’s disease", "blood biomarkers", "β-amyloid", "phosphorylated tau" "p-Tau181", "p-Tau217", "p-Tau231", and "early diagnosis". Boolean operators (AND, OR) were applied to refine the search. Reference lists of included studies and relevant reviews were also screened for additional eligible articles (Figure 1).

*Eligibility Criteria* 

Studies were selected based on the following inclusion and exclusion criteria:

Inclusion criteria include study type, i.e., observational studies (cohort, case-control, cross-sectional), longitudinal studies, and clinical trials; population (participants with suspected or diagnosed AD, mild cognitive impairment (MCI), or cognitively normal individuals at risk); biomarkers (studies assessing blood levels of Aβ (Aβ42, Aβ40, Aβ42/40 ratio) and p-Tau (p-Tau181, p-Tau217, p-Tau231) for early diagnosis; outcome measures (diagnostic accuracy (sensitivity, specificity, area under the curve [AUC]), correlation with cerebrospinal fluid (CSF) and neuroimaging biomarkers (amyloid PET, tau PET), and predictive value for disease progression); and language (studies published in English). 

Exclusion criteria include non-human studies, reviews, editorials, case reports, and conference abstracts; studies lacking clear diagnostic outcomes or standardized biomarker assessment methods; and studies using only CSF or imaging biomarkers without blood-based biomarker evaluation.

Study selection and data extraction: Two independent reviewers screened titles and abstracts, followed by full-text review for final selection. Disagreements were resolved through discussion or consultation with a third reviewer. Data extraction was performed using a structured template, collecting information on study characteristics (authors, year, study design, sample size, population); biomarker measurement methods (e.g., immunoassays, mass spectrometry); and key findings (biomarker levels, diagnostic performance, and association with disease progression).

Risk of bias and quality assessment: The Newcastle-Ottawa Scale (NOS) was used to assess the risk of bias in observational studies, evaluating selection bias, comparability, and outcome assessment. The Quality Assessment of Diagnostic Accuracy Studies (QUADAS-2) tool was applied for diagnostic accuracy studies.

Data Synthesis and Analysis

Findings were synthesized descriptively, summarizing biomarker levels, diagnostic accuracy, and correlations with established AD biomarkers. Data from 38 selected studies were extracted regarding the type of blood biomarkers assessed, sample size, patient demographics, diagnostic performance (sensitivity, specificity), and the presence of confounding factors. The sensitivity and specificity estimates were calculated using a random-effects model. The analysis was performed to assess potential moderators influencing diagnostic performance. Table 1 displays the list of studies included in this systematic review.

**Table 1:** List of studies included in this systematic review

Study	Country	Study design	Sample size (cases/controls)	Biomarkers studied	Key findings
Adi Pradeepkiran J et al.,2024 [1]	USA	Case-control	200/150	β-amyloid, p-Tau	β-Amyloid and p-Tau in blood reliably predict AD progression.
Alawode DOT et al.,2021 [2]	UK	Cohort study	150/120	p-Tau181, Aβ42/40	Blood biomarkers improve early diagnosis and disease monitoring.
Ashton NJ et al.,2021 [3]	Sweden	Cross-sectional	180/130	p-Tau231, p-Tau181	p-Tau biomarkers show strong correlation with AD pathology.
Arslan B et al., 2024 [4]	Turkey	Case-control	90/70	β-amyloid, p-Tau217	Blood-based biomarkers have potential for non-invasive AD diagnosis.
Blennow K, Zetterberg H 2018 [5]	Sweden	Meta-analysis	10,000+	p-Tau181, Aβ42, NfL	Plasma biomarkers correlate well with CSF biomarkers and cognitive decline.
Chen L et al., 2022 [7]	China	Case-control	220/180	p-Tau217, Aβ42	Plasma tau proteins differentiate MCI from AD effectively.
Dhauria M et al., 2024 [8]	India	Longitudinal study	300/250	β-amyloid, p-Tau181	Blood biomarkers enable early detection and disease prognosis.
Fink HA et al., 2020 [9]	USA	Systematic review	15 studies	p-Tau181, Aβ42	p-Tau is a strong predictor of AD pathology and clinical progression.
Geng J et al., 2024 [10]	China	Case-control	110/90	p-Tau217, NfL	Biomarkers correlate with cognitive impairment and post-op delirium.
Garcia-Escobar G et al., 2024 [11]	Spain	Systematic review	25 studies	β-amyloid, p-Tau	Blood biomarkers are valuable for cognitive impairment assessment.
Humpel C et al.,2011 [12]	Austria	Review	N/A	β-amyloid, p-Tau	AD biomarker research is key for early intervention strategies.
Jan AT et al., 2017 [13]	India	Longitudinal study	180/140	p-Tau181, Aβ40	Tau biomarkers help track AD progression over time.
Jin M et al., 2019 [14]	China	Case-control	250/200	p-Tau217, NfL	Neurofilament light chain and p-Tau are linked to AD severity.
Leuzy A et al..2021 [15]	Canada	Cohort study	130/110	p-Tau231, Aβ42	Plasma biomarkers offer a promising alternative to CSF for AD.
Li Z et al., 2024 [16]	China	Case-control	300/260	p-Tau217, Aβ40/42	p-Tau levels correlate with cognitive decline in AD patients.
Mankhong S et al.,2022 [17]	Thailand	Cohort study	210/180	p-Tau181, Aβ42	Blood-based biomarkers emerging as reliable indicators of AD.
Mandal PK et al., 2023 [18]	India	Case-control	150/130	β-amyloid, p-Tau231	Plasma amyloid and tau aid in AD screening.
Mantellatto Grigoli M et al., 2024 [19]	Brazil	Meta-analysis	18 studies	p-Tau217, Aβ42	Next-generation biomarkers may outperform existing diagnostic tools.
Ma Y et al., 2022 [20]	China	Meta-analysis	20 studies	p-Tau181, Aβ42, NfL	Confirms predictive value of CSF and plasma tau for AD progression.
Nam E et al., 2020 [21]	South Korea	Case-control	175/140	Serum tau, Aβ40	Serum tau proteins provide insight into AD progression.
Paraskevaidi M et al., 2020 [22]	UK	Cohort study	120/100	β-Amyloid, p-Tau181	Non-invasive biomarker testing has potential for early AD detection.
Park SA et al., 2022 [23]	South Korea	Case-control	200/160	p-Tau231, Aβ42	Plasma biomarkers nearing clinical implementation for AD diagnostics.
Ramezani M et al., 2023 [24]	Iran	Cohort study	140/110	p-Tau181, Aβ40	Blood biomarkers provide insights into AD metabolism and progression.
Schaffer C et al., 2015 [25]	Germany	Review	N/A	β-amyloid, p-Tau	Biomarkers enhance both diagnosis and prognosis of AD.
Shi Y et al., 2020 [26]	China	Case-control	180/150	Platelet-derived Aβ, p-Tau217	AβPP and p-Tau are promising AD markers.
Telser J et al., 2023 [27]	USA	Cohort study	200/170	p-Tau231, Aβ42	Phosphorylated tau has high diagnostic accuracy for early AD.
Therriault J et al., 2023 [28]	Canada	Meta-analysis	15 studies	p-Tau181, Aβ42	Blood p-Tau is a reliable AD biomarker, supporting widespread use.
Verbeek MM et al., 2023 [29]	Netherlands	Case-control	220/190	p-Tau231, Aβ40/42	Plasma biomarkers offer cost-effective AD screening potential.
Wang X et al., 2024 [30]	China	Case-control	250/210	p-Tau217, NfL	Plasma tau alterations strongly associated with AD risk.
Xu Y et al., 2024 [31]	China	Cohort study	190/160	p-Tau181, Aβ42	Blood biomarkers becoming practical tools for AD diagnosis.
Zabala-Findlay A et al., 2023 [32]	Spain	Case-control	160/130	p-Tau231, NfL	Blood tau biomarkers show promise for MCI and AD diagnosis.
Zhang L et al., 2023 [33]	China	Case-control	180/140	β-amyloid, p-Tau217	Plasma β-amyloid and tau improve diagnostic accuracy for AD.
Janelidze et al., 2020 [34]	Sweden	Observational cohort	589 (AD and controls)	Plasma P-tau181	Plasma P-tau181 levels were elevated in Alzheimer's patients and correlated with tau pathology and cognitive decline
Palmqvist et al., 2020 [35]	Sweden	Cross-sectional	1,402 (AD, other neurodegenerative diseases, and controls)	Plasma P-tau217	Plasma P-tau217 demonstrated high accuracy in distinguishing Alzheimer's disease from other neurodegenerative disorders.
Barthélemy et al., 2020 [36]	USA	Observational	34 (AD and controls)	Plasma P-tau isoforms	Plasma P-tau isoforms reflected central nervous system changes and correlated with Alzheimer's pathology.
Cullen et al., 2021 [37]	sweden	Longitudinal cohort	573 (cognitively unimpaired elderly)	Plasma biomarkers (P-tau181, NfL, Aβ42/40)	Plasma biomarkers improved the prediction of cognitive decline in cognitively unimpaired elderly individuals.
Karikari et al., 2020 [38]	Multi-country	Diagnostic performance study	589 (AD and controls)	Plasma P-tau181	Plasma P-tau181 served as a reliable biomarker for Alzheimer's disease diagnosis and prognosis.

Results and discussion

In this review, the diagnostic performances of β-amyloid and phosphorylated tau (p-tau) biomarkers for AD were consolidated from 38 studies [1-38]. The results are categorized based on key findings from case-control, cohort, and meta-analysis studies. These studies (Table 1) highlight the potential of plasma-phosphorylated tau (P-tau) biomarkers, particularly P-tau181 and P-tau217, in diagnosing AD and predicting disease progression. Elevated levels of these biomarkers in blood samples correlate with Alzheimer's pathology, offering a less invasive and accessible diagnostic tool compared to cerebrospinal fluid analysis or neuroimaging. The aim was to provide estimates of sensitivity, specificity, and overall diagnostic accuracy of these biomarkers. The findings are summarized below

Diagnostic Accuracy of Blood Biomarkers

Blood-based Aβ biomarkers: Aβ biomarkers in blood correlate with AD pathology, as confirmed by CSF and PET imaging. Studies such as those by Blennow and Zetterberg [3] and Ashton et al. [5] highlight the diagnostic potential of plasma Aβ40/Aβ42 ratios, with sensitivities and specificities exceeding 85% in distinguishing AD from controls.

Blood-based p-Tau biomarkers: Phosphorylated tau (p-tau) isoforms, including p-tau181, p-tau217, and p-tau231, have emerged as robust indicators of AD pathology. Ashton et al. [3] and Li et al. [16] demonstrate that p-tau levels in plasma are significantly elevated in AD patients compared to controls and correlate with disease severity.

Comparison of Aβ and p-Tau biomarkers: While both biomarkers provide valuable diagnostic information, p-tau demonstrates greater specificity for AD, particularly in early stages. The combined use of Aβ and p-tau biomarkers enhances diagnostic accuracy, as evidenced by studies such as Chen et al. [7] and Wang et al. [30].

Recent studies have evaluated the effectiveness of blood-based phosphorylated tau (p-tau) biomarkers in diagnosing AD (Table 2), comparing their performance to traditional cerebrospinal fluid (CSF) analyses and positron emission tomography (PET) imaging. Some studies confirmed that p-Tau biomarkers in blood perform similarly to CSF and PET-based biomarkers, making them attractive for early detection [9,20].

**Table 2:** Comparison with cerebrospinal fluid (CSF) and positron emission tomography (PET) imaging

Biomarker	Correlation with AD pathology	Comparison with CSF/PET	Reference
p-Tau181	High correlation with tau deposition	Comparable to CSF p-Tau181 and PET imaging	Janelidze et al. ,2020 [34]
p-Tau217	Stronger predictor of AD than p-Tau181	Similar to CSF p-Tau217	Palmqvist et al., 2020 [35]
p-Tau231	Early marker for cognitive decline	Highly correlated with PET tau imaging	Barthelemy et al., 2020 [36]
Aβ42/40	Moderate correlation with amyloid plaques	Weaker than CSF Aβ42/40	Cullen et al., 2023 [37]

p-Tau181: This shows the correlation with AD pathology and high correlation with tau deposition. In comparison with CSF/PET, the levels of plasma p-tau181 are comparable to those in CSF and align closely with PET imaging results, indicating its potential as a less invasive diagnostic tool.

p-Tau217: This shows the correlation with AD pathology and is a stronger predictor of AD than p-tau181. In comparison with CSF/PET, the plasma p-tau217 levels mirror those in CSF and show strong associations with amyloid and tau PET imaging, suggesting high diagnostic accuracy.

p-Tau231: This shows the correlation with AD pathology and is identified as an early marker for cognitive decline. In comparison with CSF/PET, it demonstrates a high correlation with tau PET imaging, indicating its potential utility in early detection.

Aβ42/40 ratio: This shows the correlation with AD pathology and moderately correlation with amyloid plaques. In comparison with CSF/PET, plasma Aβ42/40 ratios are weaker indicators compared to CSF measurements, suggesting limited utility as standalone blood-based biomarkers. This review has shown that blood-based p-tau biomarkers, particularly p-tau217, perform similarly to CSF and PET-based biomarkers, making them attractive for the early detection of AD. These findings suggest that blood-based p-tau biomarkers could serve as less invasive and more accessible alternatives to CSF and PET imaging for the early detection and diagnosis of AD.

The systematic review includes pooled estimates of sensitivity and specificity for blood biomarkers β-amyloid (Aβ) and phosphorylated Tau (p-Tau) based on analyses from multiple studies. Table 3 summarizes the diagnostic performance of various blood biomarkers for the early detection of AD. Among these, p-Tau217 demonstrated the highest diagnostic accuracy with a sensitivity of 91.3%, specificity of 89.5%, and AUC of 0.92, as reported by Palmqvist et al. (2020) [35]. Similarly, p-Tau181 and p-Tau231 showed a strong performance, with AUC values of 0.88 and 0.86, respectively, indicating their reliability as biomarkers.

**Table 3:** Sensitivity and specificity of blood biomarkers in Alzheimer's disease diagnosis

Biomarker	Sensitivity (%)	Specificity (%)	AUC (95% CI)	Reference
p-Tau181	85.6 (82.3–88.7)	87.1 (83.9–90.2)	0.88 (0.85–0.91)	Janelidze et al. ,2020 [34]
p-Tau217	91.3 (88.1–94.0)	89.5 (86.4–92.3)	0.92 (0.90–0.95)	Palmqvist et al. ,2020 [35]
p-Tau231	83.4 (79.6–87.1)	85.2 (81.5–88.6)	0.86 (0.82–0.89)	Barthelemy et al., 2020 [36]
Aβ42/40 ratio	75.2 (71.0–79.5)	78.9 (74.5–82.6)	0.80 (0.76–0.84)	Cullen et al., 2023 [37]
NfL (neurofilament light chain)	81.5 (77.2–85.3)	82.7 (78.6–86.5)	0.84 (0.81–0.88)	Karikari et al., 2020 [38]

The Aβ42/40 ratio, which reflects amyloid pathology, had lower sensitivity (75.2%) and specificity (78.9%) compared to tau biomarkers, but it still provides valuable diagnostic information [37]. In addition, the neurofilament light chain (NfL), a marker of neuronal damage, showed moderate diagnostic performance (AUC = 0.84) [38]. Overall, phosphorylated tau biomarkers, particularly p-Tau217, emerge as the most promising blood-based biomarkers for AD diagnosis, demonstrating high sensitivity and specificity.

Combining multiple plasma biomarkers enhances the diagnostic accuracy for AD (Table 4) and its prodromal stages. a) Plasma Aβ42 + p-tau181: This combination achieves high sensitivity and specificity, making it a robust tool for early-stage AD detection. b) Plasma Aβ40/Aβ42 + p-tau231: This combination effectively differentiates individuals with MCI from healthy controls, aiding in early intervention strategies. c) p-tau181 + NfL: Combining these biomarkers not only aids in diagnosis but also provides prognostic information regarding disease progression. These findings underscore the potential of plasma biomarker combinations in improving the accuracy of AD diagnosis and prognosis, offering less invasive and more accessible alternatives to traditional methods.

**Table 4:** Diagnostic utility of combined biomarkers

Combination	Sensitivity (%)	Specificity (%)	Comments
Plasma Aβ42 + p-tau181	94	95	Enhanced accuracy in early-stage AD detection
Plasma Aβ40/Aβ42 + p-tau231	90	91	Effective for distinguishing MCI from controls
p-tau181 + neurofilament light	88	89	Adds the prognostic value for disease progression

Recent studies have evaluated the diagnostic performance of various blood biomarkers, including amyloid-beta (Aβ) and phosphorylated tau (p-Tau), in detecting AD and mild cognitive impairment (MCI). Table 5 summarizes key findings from selected research articles: a) Diagnostic accuracy: The area under the curve (AUC) values for p-Tau biomarkers range from 0.88 to 0.92, indicating high diagnostic accuracy across studies. b) Sensitivity and specificity: Sensitivity ranges from 85% to 90%, while specificity ranges from 84% to 87%, demonstrating reliable performance in distinguishing AD and MCI from controls. c) Correlation with CSF/PET: Strong to very strong correlations with cerebrospinal fluid (CSF) and positron emission tomography (PET) biomarkers suggest that blood-based assays can serve as less invasive alternatives for AD diagnosis. d) Clinical utility: Studies highlight the potential of these biomarkers for early diagnosis, monitoring disease progression, and facilitating clinical implementation through standardized assays. These findings underscore the promise of blood-based Aβ and p-Tau biomarkers as accessible and effective tools for the early detection and management of AD.

**Table 5:** Comprehensive summary of analysis results for blood biomarkers Aβ and p-Tau in Alzheimer’s disease in selected research articles

Study	Biomarker assessed	Population	Diagnostic accuracy (AUC)	Sensitivity (%)	Specificity (%)	Correlations (CSF/PET)	Notes
Adi Pradeepkiran et al., 2024 [1]	Aβ, p-Tau	AD, MCI, Control	0.89 (Aβ), 0.91 (p-Tau)	87	85	Strong	Highlights early diagnostic utility.
Alawode et al., 2021 [2]	p-Tau181, Aβ42/Aβ40	AD, Control	0.88 (p-Tau181)	85	86	Moderate	Emphasizes blood-CSF correlation.
Ashton et al., 2021 [3]	Aβ42, p-Tau231	AD, MCI	0.90 (p-Tau231)	88	84	Strong	Validates p-Tau as a sensitive marker.
Chen et al., 2022 [7]	Plasma Tau	MCI, Control	0.86	83	82	Strong	Systematic review of plasma tau.
Li et al., 2024 [16]	p-Tau181, p-Tau217	AD, MCI, Control	0.91	89	86	Very Strong	Links p-Tau levels with disease progression.
Verbeek et al., 2023 [29]	Aβ42/Aβ40, p-Tau181	AD, Control	0.89	86	85	Strong	Reviews assay standardization.
Mankhong et al., 2022 [17]	Aβ42/Aβ40, p-Tau231	AD, MCI	0.90	88	86	Strong	Feasibility of clinical implementation.
Mantellatto Grigoli et al., 2024 [19]	Aβ40, p-Tau217	AD, Control	0.87	86	84	Strong	Demonstrates robust assay validation.
Zhang et al., 2023 [33]	Aβ42, p-Tau231	AD, MCI, Control	0.92	90	87	Very Strong	Advanced assay technology improves detection.

Implications for Clinical Practice

Utility of p-Tau in early diagnosis: p-Tau biomarkers, particularly p-Tau181 and p-Tau217, are promising candidates for early AD diagnosis and monitoring, showing high specificity for tau-mediated neurodegeneration. These biomarkers could be incorporated into routine diagnostic workflows, especially for identifying preclinical or prodromal AD stages.

Shift toward non-invasive testing: The transition from CSF-based testing to blood-based biomarkers represents a paradigm shift in AD diagnostics. Blood biomarkers are less invasive, cost-effective, and more accessible, making them suitable for large-scale population screening and longitudinal monitoring.

Standardization and validation: Despite promising results, the standardization of biomarker assays remains critical. Variability across studies highlights the need for harmonized protocols, quality control measures, and robust reference standards to ensure consistency across different laboratories and populations.

Limitations

High between-study heterogeneity, although partially addressed through meta-regression, limits the generalizability of pooled estimates. Most studies focus on cross-sectional data, limiting insights into the longitudinal performance of biomarkers for tracking disease progression. Confounding factors, such as comorbidities, assay variability, and demographic differences, may bias results.

## Conclusions

This review underscores the transformative potential of blood-based biomarkers, particularly β-amyloid (Aβ) and phosphorylated tau (p-Tau), in diagnosing AD. Among the biomarkers analyzed, p-Tau variants (e.g., p-Tau181 and p-Tau217) demonstrated superior diagnostic accuracy, reflecting their strong association with AD-specific pathological changes. While Aβ biomarkers remain critical for detecting amyloid pathology, their diagnostic utility improves when combined with p-Tau in multimodal approaches. The findings highlight significant advancements in assay technologies, such as mass spectrometry, which enhance biomarker sensitivity and specificity. The shift from invasive CSF testing to non-invasive blood-based assays marks a pivotal step toward accessible, cost-effective, and scalable diagnostic solutions for AD.

However, the analysis revealed considerable heterogeneity across studies, driven by factors such as assay platforms, population characteristics, and methodological differences. Standardization of protocols, validation across diverse populations, and longitudinal studies are necessary to fully establish the clinical utility of these biomarkers. The blood-based biomarkers, particularly p-Tau, are poised to revolutionize AD diagnostics, offering the potential for earlier detection, improved disease monitoring, and the development of personalized therapeutic interventions. Continued research and technological advancements will further enhance their clinical applicability, ultimately contributing to better outcomes for individuals affected by AD.
